# Dr. Hird, on the Influenza

**Published:** 1803-12-01

**Authors:** Thomas Beddoes


### To the Editors of the Medical and Physical Journal.

GENTLEMEN,

THE accompanying Communications supply some important dates and circumstances towards the history of the Influenza; I cannot now expect to receive any more, and therefore shall trouble you, against your next Number, with my own Remarks, in which I shall introduce a very satisfactory Report from Dr. Carter of Canterbury, on the appearance of this complaint in Kent, together with one or two others.

The first of the following numbers relates to the district about York, and would have been transmitted with the last parcel, had it arrived in time.

I am, &c.

14 Nov. 1803. THOMAS BEDDOES.

---

112. Mr. BELL, Pocklington, Oct. 8, 1803.

The first patients I had attacked with the Influenza were about the middle of March, with febrile disease, cough, and more or less painful inflammation in the lungs and pleura, the removal of which was not difficult by bleeding, saline antimonial nitrous remedies, and demulcents for the cough; in short, I then considered them as the usual spring complaints of peripneumony and pleurisy, which diseases occur frequently with us in the months of January, February, and March. In the beginning of April, a remarkable number of patients, of all ages, were attacked, the middle aged and robust most severely; and of the whole number, I lost one aged patient and two infants; the rest all recovered well under the above treatment, and I do not remember one case that terminated in hectic fever or pulmonary suppuration; though from such species of disorders, on other occasions, I have often had cause to observe and lament it. I confess I did not, at the beginning or in its progress, consider it contagious, although whole families were seized; I supposed exposure to the same circumstances of atmosphere might occasion similar attacks, and I looked on it as endemial but not epidemical, putrid symptoms hardly ever occurring, and then only slight diarrhœas in weak habits, the principal symptoms always appearing purely inflammatory, and very few cases of long duration, in a few days the inflammation always being checked, and expectoration soon relieved the cough. —Not being in the habit of taking notes except in some particular circumstances, my day book and memory, with the assistance of my young man, are what I write from; but if you have any more particular questions, I will with pleasure answer them. The disease began to decline the latter end of May, and was nearly ended by the middle of June.

113. Dr. Hird, Leeds.

The following particulars I took down from the mouth of Dr. H. who, when I saw him here, could not specify the time of its appearance at Leeds; but I have reason to hope he will supply the date soon.

The influenza did not spread rapidly at first; for a week or ten days it seemed to abate, and then it became very general. It shewed itself by great and sudden debility, pain in the head, rigours, heats, and perspirations. The cough was very constant; it followed pain in the head and shivering, but seldom or never, in my observation, preceded those symptoms. The bowels, in some persons, were affected with pain and diarrhœa. Females seemed to be more liable to its attack than males; and the delicate among females more liable than the robust. Few phthisical females escaped it; those of sedentary habits were more universally affected. Men, who lived free or full, were less liable; and such as went very much out of doors with such a diet, were least of all liable.

My usual practice was to give first an emetic, then the effervescent saline draught, and extract of poppy when the cough became very troublesome; this appeared preferable to opium itself. When every inflammatory symptom had gone off, the spt. ætheris vitr. c. with white poppy syrup, was useful. Old asthmatics died; and it was speedily over with them if they had been bled or otherwise debilitated. Bleeding with leeches was singularly useful in robuster subjects, where there was pain in the side, &c.

Four out of seven in Dr. H's family had it at the same time. Of bed-fellows, sometimes one had it, and one had it not. In a particular family that resided in an insulated house it was universal, and all were seized within thirty-six hours; the family consisted of either fourteen or fifteen. In other instances, it seemed as if propagated from person to person.

114. Dr.

114. Dr. Rutter, Liverpool, Oct. 31, 1803.

I think I met with an instance of the influenza on the 27th of February, 1803. My patient was then in the second day of the disease, which at first appeared to be a combination of rheumatism and catarrh. The pains, which were betwixt the false ribs and the spine of the ileum, were uncommonly severe, and resembled those of rheumatism; these, however, gave way in a day or two, and left a catarrhal affection attended with great debility, exactly resembling the influenza which afterwards became so general. On the 5th of March I saw a strongly-marked case of the influenza; and the patient, a young lady, was then in the fifth day of the disease. In the second and third week of March, the influenza was very general here; towards the middle of April it had greatly abated; and before the end of that month, as far as my knowledge and recollection extend, it had nearly if not entirely disappeared.

It will be unnecessary for me to swell this letter with a detailed description of the disease; in its general features, it corresponded with all the accounts which have been published; I shall only mention some particular symptoms which have not been generally noticed by other gentlemen. In many instances the disease came on with more or less of syncope; in these cases, persons were seized in a state of apparent good health with great faintness, and some fainted away; and in this manner I have known the attack to take place in some robust labouring men: then followed chillness, shivering, head-ach, pains in the head, loins, and limbs, cough, dyspnœa, soreness and stitches in the chest, &c. Soreness in the throat, as it was called, was a constant symptom in all the cases which I saw. The patients referred this soreness to the larynx or upper part of the trachea; and in some cases, the soreness distinctly and gradually diffused itself from this point, through the trachea and the ramifications of the bronchæ, until the whole chest was affected with it; but it was felt most severely under the sternum, along its whole length, especially on coughing or inspiring. The dyspnœa was in general very laborious, and was frequently attended with sharp stitches in the chest. One of my patients who had the disease very severely, and whose breathing was extremely laborious, told me that she felt as if she were breathing through a sponge. The cough was mostly dry at first, and was very harrassing. When expectoration took place, it was in some cases very profuse, and in two or three instances I observed it slightly tinged with blood. The pulse in every case which I saw, was soft and small, though frequent. I did not meet with one case with which pneumonic inflammation was combined in such a degree as to authorize the use of the lancet. The disease was not very fatal here.

I have nothing material to add with respect to the treatment. Emetics were very useful at the commencement of the disease; and when followed with a smart dose of calomel or Castor oil, checked the further progress of it in some instances. It was of great advantage throughout the disease to keep the bowels regular. When the disease was formed, the dyspnœa and stitches, as well as the soreness in the chest, were relieved most effectually by blisters, which it was sometimes necessary to repeat before these symptoms gave way. In circumstances indicating great oppression of the chest, the lancet was prohibited by the uncommon prostration of strength, and by the softness and smallness of the pulse. In some instances the debility was so considerable, and so great was the tendency to syncope on being raised up in bed, that it was necessary to throw in mild nourishment with as much assiduity as in the debility of typhus; the respiration at the same time being difficult and laborious, and the cough rending and incessant. After a gentle diaphoresis was produced by antimonials and diluents, no advantage was obtained by keeping the patients longer in bed; on the contrary, those seemed to regain their strength more speedily, who, notwithstanding their debility, endeavoured to get up, and to remain out of bed during some part of each day. Opiates were hurtful in the commencement of the disease; they increased the febrile heat, and retarded the advancement of expectoration; in the decline of the disease, they were highly beneficial.

This is all that occurs to me on the subject. I took no memorandum of the disease, except relating to the date of its appearance. If you think the preceding remarks of any value, they are at your service. It is useless now to lament that I have not been more particular in my observations, or that I did not record them at the time; the want of such a record must therefore be my apology for sending you such a barren fragment.

115. Mr. Whateley, Burton on Trent, October 2, 1803.

In my practice I did not meet with any instance of disease the last spring that could be denominated influenza or catarrh catarrh arising from human contagion, nor were a greater proportion of persons indisposed than usual at that season. The scarlatina of the mildest kind had been for some time very frequent in the town and neighbourhood, and the measles and hooping cough were common last spring with generally favourable symptoms. I feel satisfied the disorder, which was so prevalent in most parts of the kingdom early in the year, was never met with in this place or its immediate vicinity, at least not commonly.

116. Mr. Ward, Woodchester, Nov. 7, 1803.

There being a great inconsonancy of opinion respecting the disease lately prevalent, whether contagious or not, I take the liberty of troubling you with a few observations I had an opportunity of making in my practice. I cannot flatter myself they will tend to elucidate the decision of so important a question, but should they be found worthy of a place among your numerous and valuable collection of communications on the same subject, I have to acknowledge it with gratitude.

The first case characterising the disease denominated influenza appeared here on the 20th of March. (The last case in the beginning of May.) In the ensuing week I visited eight persons, who from the severity of their attack were necessitated to keep their beds, independent of *many* who from the lenity of their symptoms were exempt from such confinement.

Some who were attacked with this disease, evidenced symptoms closely allied to typhus. When the pulse from diminished energy indicated the necessity of avoiding profuse evacuations, I found no medicine so effectual as the mist. camph. aq. ammon. acet. & vin. ant. tart.

In three cases where the symptoms were more purely inflammatory, accompanied with pneumonia, catarrh, &c. I did not hesitate using the lancet; the relief obtained fully sanctioned this unapproved treatment. In those cases nothing appeared to me of greater and more conspicuous advantage than frequent nauseating doses of the vin. ant. tart. alternating the saline mixture in a state of effervescence. Blisters did not appear to me of the least service; in two or three cases I thought they aggravated the disease. Several patients, whose attack was not violent, were wholly relieved by profuse perspiration, I found the pulv. ipecac. c. fully competent to this purpose and apparently a preventive of the further progress of the disease. A respectable clothier's son in this neighbourhood, whose attack was alarmingly alarmingly severe, was greatly relieved by an eruption on the skin, which disappearing, a recurrence of the symptoms took place. A chlorotic girl, where evacuations were cautiously avoided, became anasarcous on the departure of the influenza. With women in their accouchement, I had an opportunity of seeing two cases; they were fortunately not violent; the secretion of milk was almost wholly interrupted; they were both relieved by the pulv. ipecac. c. The important question, whether the influenza was contagious or not, I leave to the decision of those able practitioners whose opinions will be influenced solely by facts, collected from candid and impartial observations; at present, till conviction of premature and mistaken judgment convert me to opposite sentiments, I consider it as contagious, and primarily originating in an observable difference in the atmosphere many days preceeding the appearance of the disease.

A family (seven in number) with whom I am particularly intimate, were apprehensive of taking the infection from my frequent visits; their expectations were realized, and not one escaped the infection. At this time I participated with my friends, though, fortunately, our attack was very mild.

117. Mr. Brewer, Newport, Monmouthshire, October 23, 1803.

I should have been particularly happy in giving you any satisfactory account of the influenza which appeared here about the 20th of February, under the same form that it did in most other places, of which there has been so much already said, that I can do very little more than give my opinion contrary to its contagious effects. My reasons for which are—Its appearing first in the most retired part of this country, and in a family which, from the strictest enquiry, could have no communication with any source of infection.—Its attacking large families at the same instant almost, that is, eight or ten in the course of two days; and some of those not able to account for any communication with the disease.—And its attacking one of two who lay in the same bed, while the other remained perfectly free from the disease; and this in three or four instances within my knowledge. The complaint was in many cases very severe, but fatal to very few. I saw but one who died immediately of the epidemic, a lady far advanced in years and long troubled with asthma. I believe it has hastened the termination of many previous affections of the chest, and has also laid the foundation of many others that will terminate seriously. The last attack of the disease which occurred to me was on the 15th of April.

118. Mr. Pew, Shaftesbury, Nov. 4, 1803.

Although I did not see occasion to record any thing in the late influenza, (which appeared in a much milder form than that which occurred, I think, in 1779, in as much as it left no permanent weakness, no loss of nervous or muscular power behind it) yet to shew my readiness to comply with any request of your's, I shall state from memory the general symptoms. It commenced as it were suddenly and pretty generally the first week in April, so that I believe most people hereabout became affected from the influence of the atmosphere, although I have little doubt of its truly infectious nature, especially when concentrated by a number of *maladies* in large cities. It was generally attended by catarrhal symptoms, sometimes more, sometimes less severe, often with pain in the side, always with great prostration of strength, and I believe in every instance with a very disagreeable sense of fullness and pain over the eyes, and a sensation of pressure as it were on the ball itself. No case occurred in my own practice in which I thought it prudent to bleed, but I had occasion to see two cases in which the practitioner had recourse to that evacuation; the blood was not sizy, and I am in doubt whether it was of advantage, but it did not seem to retard the return of strength; with me the pulv. ipecac. c. gr. v. 4ta. *quaque hora* seemed to produce very good effects. I think the complaint nearly left us by the latter end of the month. I have no record of the rate of the pulse.

I had almost forgot to mention that a few feeble old people were carried off apoplectic, excited by the influenzal poison.

119. Dr. Callanan, Cork, Oct. 1, 1803.

I think I cannot answer the queries with which you favoured me by Mr. Beaufort, in a more unbiassed manner than by sending you a copy of the remarks on the influenza, which occurred to me during its progress.

As to your first query, (respecting its contagious nature) I must answer positively in the affirmative, for the reasons advanced in the remarks, and from what I had seen in the influenza of 1780. The lady who infected me (sit verbo venia) was one of the first who had it in Cork. She has lately traced traced its genealogy to me, by telling me, "She got it by sitting at table next to a gentleman just arrived from Dublin, where the disorder raged universally." I remember our being free from it in town by the time it had reached the remote parts of the country; even the difference of a week at not twelve miles distance where the communication with town was not so frequent, as in the late epidemic when the play-house and assizes brought town and country more in contact. Innumerable instances of individuals transferring the contagion can be cited on the best authority, and most unprejudiced evidence; the gentleman who kept the school in the Great Island, in his letters to me, for three weeks triumphed in the idea of his escaping; but, as I predicted, he suffered in his turn.

2d. I never saw an instance of such catarrhs originating from fomes, nor do I know that any has been so well traced to its origin as that of 1780. Maertens says, that at Petersburgh, on a cold night, the thermometer rose 30° of Fahrenheit; the next morning forty thousand people were taken ill with the influenza, thence it over-ran Europe progressively.

3d. In the beginning of the epidemic, I have been mistaken, as I mentioned; the symptoms are certainly for the most part catarrhal; it may probably happen that those who have the disorder mildly are considered only to have a common cold; and, vice versa, a heavy accidental catarrh may rank as influenza; but in the latter case it would be difficult for a patient, so affected and exposed to contagion, to escape its effects, and not have a complication or an aggravation of one disorder by the other. To those who doubt of the contagious nature of the influenza, it may be a sufficient clue to judge from the prevalence of the complaint in the place, and the leading symptoms enumerated in the remarks; these speculations are liable to many objections, but cannot be attended with any bad consequences in practice.

4th. This question will suggest similar doubts in drawing conclusions; it appeared to me that Sydenham's idea was well founded, "that these leviathans absorbed all the lesser epidemics;" to a certainty, modified them prodigiously.

5th. Has been anticipated in the remarks, also the 6th and 7th.

You may be assured, Sir, that, though I am conscious I may have deceived myself, I would not disguise the truth, such as it appeared to me, or lead you into error by a false report. I am also confirmed, as yet, in my conceptions of the the subject by the co-incidence of my fellow practitioners in these quarters, who all have the same opinions both as to facts and theory.

*Remarks referred to in the above Letter.*

I did not see any case of influenza, although in daily expectation of its arrival, until the 27th of March 1803, when I visited a gentleman who had for some years past three or four violent attacks of hæmoptœ. It began with symptoms of pleurisy, and was treated with bleeding to ℥x, antimonials, &c.; the pleuritic symptoms did not yield until the 7th day; the loss of appetite and peculiar creaminess of the tongue remained to the 14th day; whether the slow recovery was owing to the bleeding, I cannot conclude, as it was the only instance in which I prescribed V. S. A gentleman of the ~~faculty~~, subject to hæmoptysis, bled himself on the first attack without any seeming disadvantage. I have reason to believe that bleeding, pushed to any length, has been injurious in this epidemic.

This and the second patient, on the 28th, were attacked nearly in the same manner, and attributed, as I did, their complaints to cold caught in a crowded Court-house. On the succeeding days such numbers were taken ill, there remained no doubt on the nature of the disorder. Its course was evidently traced from England to Waterford, where it raged at the assizes, before the assizes of Cork. The assemblage in courts, with the assistance of the theatre, disseminated the contagion through town and country more rapidly than any epidemic I ever saw.

That it was communicated from person to person there can be no possibility of doubting, as its progress from the continent can be evidently marked; its appearing first in great towns most connected with the capital, and its raging some weeks later in the more distant parts of the country when it had exhausted itself in the cities. Numberless instances can be quoted to prove this assertion; amongst many others, a boarding-school truly insulated in the Great Island near Cove, remained uninfected for three or four weeks during the reign of the disorder in Cork, until a boy went from town, sickened, and infected the whole family; Mr. Alby's school, four miles from Cork, is said to have escaped. The disorder did not penetrate into the parishes of Baltimore, Cape Clear, and that part of the sea-coast, although Ross, Cloghnalkilty, and that neighbourhood, suffered.

The contagion, on many occasions, shewed itself the day after after exposure. It was early introduced into the Convent at Cork, a boarding-school, where the inhabitants may amount to about 70; four or five were generally taken ill every day, besides those who were either affected slightly or seemed to escape.

No greater variety can exist in any disorder than in its mode of attack, which in some was violent with the usual symptoms of fever; these, in general, were soonest well, probably from submitting instantly to proper treatment; others scarcely felt any indisposition, but most a considerable defluxion from the nose and eyes, with a very peculiar confusion in the head; and after the first day a singular creamy appearance on the tongue, continuing for several days, and being a sure index of the disorder still existing, where from other symptoms it may not be suspected; the cough in many, but not in all, was extremely distressing, the fits continuing a long time in the most harrassing manner. It was attended, more than any disorder I ever met, with a degree of debility and enervation scarcely to be conceived, which in many remained for a long time; in the country, some strong athletic peasants fainted on its commencement; some have never totally recovered their former strength. An unusual enormous pain in the back and loins, extending down the back part of the thighs, was not uncommon, and remained for a week or two after recovery; restlessness was a frequent and distressing symptom, which was relieved more effectually by camphor than by opium; others were affected with deafness, few with ophthalmia, and not many with diarrhœa.

The consumptive were great sufferers by it, the asthmatic and the aged; several of this class died, particularly such as were reduced in flesh and strength; some almost suddenly and unexpectedly. Dr. F. aged 77, had it four days, and imagining himself well, walked out about 200 yards from home; he fell almost apoplectic, and nearly perished in attempting to walk back a few steps; he was taken home in a sedan; has recovered his strength but slowly. Mrs. W. a feeble old lady, nearly sunk under its first attack. I have not seen an instance of its producing sudden dissolution, but from these instances believe it possible.

Its most remarkable character was its renewing every complaint latent in the constitution and affecting the weak parts; independent of pulmonary complaints, which it grievously exasperated, I have known it renew every disorder of the nerves; a paralytic affection of the leg; a singular anomalous lethargy; deafness; uterine profuse evacuevacuations; gout; a slight apoplexy; melancholia and two cases of mania in predisposed persons. Several cases of hemicrania; some agues, easily yielding to the bark; a very extraordinary case of fits of suffocation, threatening immediate dissolution, which was cured by blisters, antispasmodics and diuretics.

No one died of the simple influenza; a few only of complication with pulmonary complaints; and a few of the aged and infirm. Children, even infants, were attacked by it, of whom I hear a few perished.

The disorder at first appeared tractable, especially while the weather was mild, until the 8th of April. The cold which succeeded, determined it more to the lungs, and caused some relapses; taking an antimonial at the commencement, confinement, and perspiration, generally took down the fever in 48 hours, and gave room for nourishment and free air; many, however, suffered by too sudden exposure to cold, and relapsed. The antimonial course did not seem to succeed beyond the third or fourth day; at that period succeeded so great a state of debility as required wine, light food, and a gradual return to full living; this was so evident in the great majority that a contrary treatment must have been destructive; effervescing mixtures were of use in removing the bad taste and whiteness of the tongue, which often remained for a long time.

The disorder became rare on the 20th of April; the last case I visited was on the 29th of April.

120. Dr. Gibney, of Navan, Ireland, writes, that after the influenza, a low fever, almost constantly prevailing in that town, disappeared for a considerable time.—This is quoted from memory, his letter having been unfortunately mislaid.

121. Mr. Stanley, Drogheda, Ireland, Sept. 30, 1803.

I had the favour to receive your letter this morning, desiring to be informed at what time the influenza made its appearance in Drogheda and its environs. On recollection, I think as near as I can remember, the first appearance of this complaint took place in Drogheda early in the month of March last, and towards the end of that month became epidemic, and appeared in general amongst the people; at the same time it prevailed all through the county of Meath, and it continued uniformly through April and to the end of May, at least till the weather set in warm and dry, at which time it seemed to cease, and its progress to stop stop. I am happy to have it in my power to give you this information.

122. Dr. Barnside, Sligo, Ireland, October 21, 1803.

I find, on referring to my note book, that the first cases of influenza which came under my care occurred on the 30th of April last. The disease might have appeared two or three days earlier, as I happened to be absent in the country; I am however fully satisfied, that it did not in any instance take place before the last week in April. In Mayo, the next county, and to the westward of this, it did not appear until the latter end of the first or beginning of the second week in May. Having heard that the disease prevailed along the eastern coast of this kingdom for some weeks before, I was fully prepared to expect it here, so that you may rest satisfied I am accurate as to the time of its first appearance; and that it ceased in less than three weeks, having affected at least five-sixths of the inhabitants of this town and neighbourhood.

In a great majority of cases the attack was so slight, that the patients did not feel it necessary to go to bed, or use any remedy; in others, where it commenced with more severity, an emetic, followed by profuse perspiration, put a period to the complaint in less than 24 hours.

In the worst cases the general symptoms were, sudden and severe pain in the head and small of the back; great languor and disposition to sleep; cough; soreness of the chest, and difficult expectoration of mucus, occasionally tinged with blood; thirst; the pulse seldom became very quick, never that I could observe hard, but full, soft, and bounding; the tongue was loaded with a loose, thick, white, and curdy-like fur, which frequently remained for some time after the pulse came down to the natural standard. A few cases were attended with slight stitches, but in no instance was much difficulty of breathing observed; delirium occurred only in one case.

The appearance of the tongue, and soft bounding feel of the pulse, were symptoms so peculiar, as readily marked the difference between this and any other catarrhal fever I had ever seen. After the disease had gone off, the symptoms were likely to recur, if the patient indulged too early in getting out of bed, or in eating animal food, and drinking wine.

The duration of the disease was altogether uncertain, from one to twelve days; and it appeared most severe in those those advanced in life, or where any disease or delicacy of the pulmonary system existed.

Every patient that came under my care (and the number was very great) in this disease recovered. On the first day I gave an emetic, opened the bowels, and afterwards ordered ammonia acetat. with vin. ant. to produce perspiration. If severe cough and difficult expectoration came on, a blister was applied to the chest, and the mist. muscilag. with tinct. opii was given with seeming advantage. When the fever lasted many days, I observed decided advantage to follow smart purgatives of calomel and jalap. or pulv. antimon. I never considered the lancet necessary in this disease; I have heard that it had been used in some cases which terminated fatally: but I know that blood-letting had been used, and in some instances repeated, in the newly raised regiment of Sligo militia, and that the patients did recover.

I have shortly detailed the most important circumstances which took place in influenza; should I in any degree add to or confirm the information you have already received, I shall feel happy, convinced that whatever you acquire, will be freely communicated for the public advantage.

123. I addressed myself to the Rev. Mr. Carey, Guernsey; particularly wishing to know if there were any facts, indicating whether the influenza had arrived thither from England or France. Mr. C. informed me that he had applied to the faculty there, but that no such disorder had been observed in the island.

I have not yet succeeded in procuring information from Jersey.

124. Dr. Scott, Isle of Man, has favoured me with a copy of his detailed communication to Dr. Duncan, by whom it will doubtless be published in the Annals of Medicine. I shall only permit myself to transcribe the first paragraph.

"The influenza," says Dr. Scott, "appeared among us towards the end of March; the first patient I saw was on the 24th; he received the infection from a gentleman, who two days before had arrived from Parkgate, and had been seized with the complaint in London, and was still labouring under it. In a few days one of our Liverpool packets arrived, having many patients under the epidemic. From my enquiries I have every reason to think that thus it was imported among us."

